# Position of Patients during the Lying-in-Period

**Published:** 1890-02

**Authors:** 


### Selections and Abstracts.

Position of Patients During the Lying-in-Period.—Dr. Cullingworth in an address before the British Medical Association recommended, that lying-in-women be required to sit upright in bed while nursing, and to kneel while passing water. His reasons where, that it prevented stagnation of the locia, the avoidance of constipation and retention of urine and promoted uterine contraction.

